# Efficacy and safety of anti-PD-1 antibody plus chemoradiotherapy in locally advanced esophageal squamous cancer

**DOI:** 10.3389/fonc.2023.1005856

**Published:** 2023-02-09

**Authors:** Ji Ma, Nan Yao, Jiaying Lu, Wanxi Qu, Li Cui, Shiwang Yuan, Na Li, Shaodong Tong, Zhaohui Qin, Yuanhu Yao

**Affiliations:** ^1^ Department of Radiation Oncology, The Affiliated Hospital of Xuzhou Medical University, Xuzhou, Jiangsu, China; ^2^ Department of Radiation Oncology, Xuzhou Central Hospital, Xuzhou, Jiangsu, China; ^3^ Department of Radiation Oncology, The Third People’s Hospital of Xuzhou, Xuzhou, Jiangsu, China; ^4^ Research Center for Medical and Health Emergency Rescue, Xuzhou Medical University, Xuzhou, Jiangsu, China; ^5^ School of Medical Imaging, Xuzhou Medical University, Xuzhou, Jiangsu, China

**Keywords:** anti-PD-1 antibodies, chemoradiotherapy, esophageal squamous cell carcinoma, progression-free survival (PFS), overall survival (OS)

## Abstract

**Objective:**

To compare effects and adverse events of anti-programmed cell death protein 1 (anti-PD-1) antibody combined with chemoradiotherapy (CRT) and CRT alone as the initial treatment in locally advanced esophageal squamous cell carcinoma (ESCC).

**Methods:**

We retrospectively reviewed locally advanced ESCC patients who received Anti-PD-1+CRT as initial treatment at 3 institutions. Primary outcomes of interest were progression-free survival (PFS) and overall survival (OS); secondary outcomes were objective response rate (ORR), disease control rate (DCR), duration of response (DoR), and treatment-related adverse events (AEs) including immune-related adverse events (irAEs).

**Results:**

At data cutoff, 81 patients were included (30 Anti-PD-1+CRT, 51 CRT). Median follow-up was 31.4 months. Anti-PD-1+CRT resulted in significant improvements in PFS (median, 18.6 *vs.* 11.8 months, HR 0.48 [95% CI, 0.29–0.80], P = 0.008), and OS (median, 27.7 *vs.* 17.4 months, HR 0.37 [95% CI, 0.22–0.63], P = 0.002), compared with CRT in ESCC. The ORR and DCR of patients treated with Anti-PD-1+CRT were also significantly higher than those treated with CRT (80.0% *vs.* 56.9%, P = 0.034), (100% *vs.* 82.4%, P = 0.023), respectively. Anti-PD-1+CRT had better durable response compared with CRT, with DoR (median,17.3 *vs.* 11.1 months, P = 0.022). Treatment-related adverse event incidence was similar between the two groups (any Grade, 93.3% *vs.* 92.2%; ≥Grade 3, 50.0% *vs.* 33.3%).

**Conclusion:**

Anti-PD-1 plus chemoradiotherapy demonstrated promising antitumor activity and was well tolerated in locally advanced ESCC.

## Introduction

Esophageal cancer is the seventh most frequently diagnosed cancer (3.1%), as well as the sixth most common cause of cancer death (5.5%) in global cancer statistics 2020 (1). There is no other country with a higher incidence of esophageal cancer than China, it accounts for 50% of global morbidity and mortality, and esophageal squamous cell carcinoma (ESCC) is the primary histological type (about 80%) (2, 3).

In ESCC, radical surgery is the preferred treatment. However, 50–60% of patients are already in the advanced stages of the disease upon admission and miss the opportunity for radical resection, with a 5-year survival rate of only 20–30% (4, 5). Definitive chemoradiotherapy (CRT) with platinum-based chemotherapy in combination with taxanes or fluoropyrimidine is considered to be the standard treatment for locally advanced ESCC (6–8). In spite of treatment with the current standard of care of CRT, survival outcomes remain suboptimal, and almost all patients eventually suffer from tumor progression (9, 10). Given the prevalence and dismal survival outcomes of locally advanced esophageal cancer, clinical investigators have been working tirelessly to develop new interventions and combination therapies to prolong survival. Regretfully, these efforts to optimize treatment outcomes through anti-epidermal growth factor receptor (EGFR) antibody combing with CRT have failed so far (11, 12).

In recent years, immune checkpoint inhibitors (ICIs) have made new breakthroughs in various tumor types, including combination therapy for esophageal cancer. According to the results of the KEYNOTE-590 and CheckMate-648 studies, the US Food and Drug Administration (FDA) approved pembrolizumab and nivolumab combined with chemotherapy as a first-line treatment for advanced esophageal cancer (13, 14). In the area of neoadjuvant therapy, CheckMate-577 phase III study and a phase II study (NECT 02844075) also showed the feasibility of ICIs combined with chemoradiotherapy (15, 16). Given this background, the study was designed to evaluate the safety and antitumor activity of the initial therapy with anti-PD-1 plus CRT, which is the most widely used treatment in China for locally advanced ESCC.

## Materials and methods

### Patients

**Figure 1:**
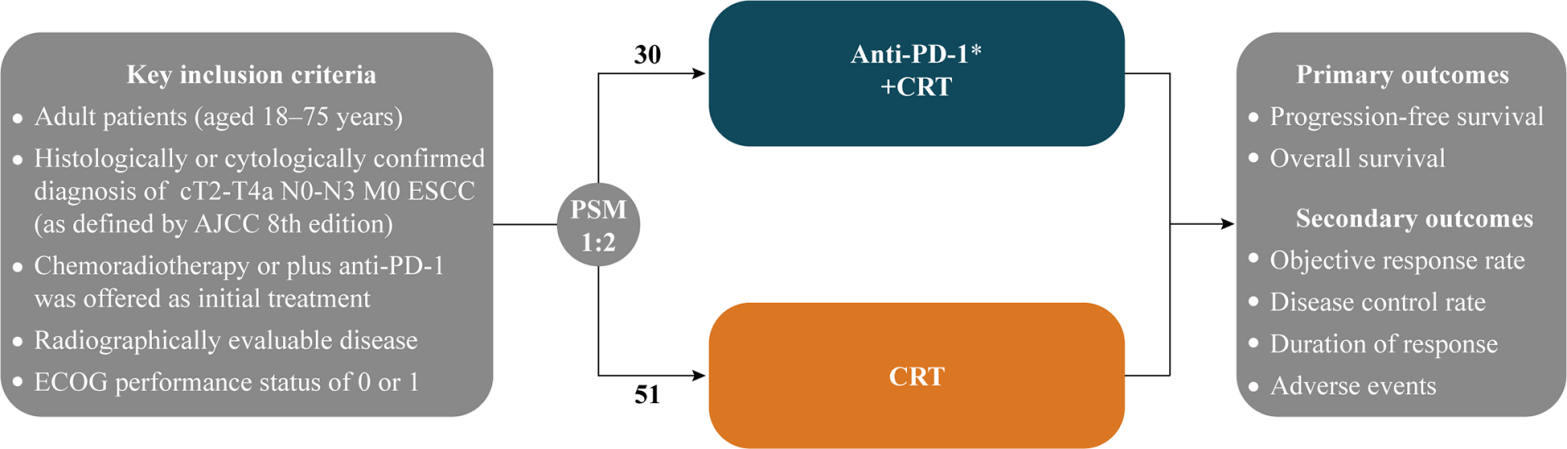
Maintain or until investigator-assessed disease progression, unacceptable toxicity, or withdrawal for other reasons; ESCC, Esophageal squamous cell carcinoma; AJCC, American Joint Committee on Cancer; ECOG, Eastern Cooperative Oncology Group; PSM: 1:2 nearest neighbor-matching with a caliper of 0.3; CRT, Chemoradiotherapy.

Clinical data for the study were collected from the Affiliated Hospital of Xuzhou Medical University, Xuzhou Central Hospital, and the Third People’s Hospital of Xuzhou (China) between January 2019 and June 2022. Inclusion criteria: 1) histologically or cytologically confirmed localized ESCC, stage II-IVa (American Joint Committee on Cancer 8th edition) nonoperative ESCC (medically unresectable or patient refused to undergo surgery); 2) chemoradiotherapy or plus anti-PD-1 was offered as initial treatment; 3) adult patients (aged 18–75 years) and Eastern Cooperative Oncology Group (ECOG) performance status of 0 or 1; 4) none of the patients received chemotherapy, radiotherapy, targeted therapies or other immune-oncology therapies prior to initial treatment (**Figure 1**).

### Treatment protocol

#### Immunotherapy

Immunotherapy was administered concurrently with chemoradiotherapy, and 200 mg of PD-1 inhibitors (tislelizumab, camrelizumab, pembrolizumab) was administered intravenously (IV) on day 1 of each 3-week cycle until progression of the disease or unacceptable toxicity occurred.

#### Chemoradiotherapy

All patients received standard intensity-modulated radiotherapy (IMRT), a dose of 50.4–66.0 Gy (1.8–2.0 Gy/fraction, 5 times/week). Chemotherapy started on the first day of radiotherapy, which consisted of paclitaxel or fluorouracil (or its derivatives) combined with platinum-containing dual drugs every 3 weeks for 2–4 cycles. The common chemotherapy regimens included TP (paclitaxel [135 mg/m^2^ IV on day 1] plus cisplatin [25 mg/m^2^ IV on days 1–3] or carboplatin [AUC = 5 IV on day 1], 3-week cycle); FP (5-fluorouracil [750–1000 mg/m^2^ continuous IV on days 1–4] plus cisplatin [25 mg/m^2^ IV on days 1–3], 4-week cycle).

### Endpoints and clinical evaluation

In this study, the primary outcomes were overall survival (OS) and progression-free survival (PFS); secondary outcomes were objective response rate (ORR), disease control rate (DCR), duration of response (DoR) and treatment-related adverse events (AEs) including immune-related adverse events (irAEs). Investigators evaluated clinical response according to Response Evaluation Criteria in Solid Tumors version 1.1 (RECIST 1.1) or immune-related RECIST (irRECIST) and Ren et al. ‘s research (17, 18) (**Supplementary Tables 1**-**3**). Tumor imaging and assessment of disease were performed by computed tomography (CT) within 2 weeks before treatment and 4-6 weeks after the last dose of radiotherapy, and every 3 months (3 or 4 cycles) thereafter. Follow-up assessments included clinical physical examinations, routine hematological and biochemistry tests, esophagogram, endoscopic ultrasonography, thorax and upper abdomen CT, magnetic resonance imaging, and positron emission tomography-CT. Telephone follow-up was performed for patients who were lost to regular medical follow-up records before death. Safety and tolerability included adverse events (AEs), serious AEs (grade 3 or higher AEs) were evaluated according to NCI common terminology for adverse events version 5.0 (NCI-CTCAE v5.0), and irAEs (immune-related AEs) were assessed using peer-reviewed irAEs management guidelines (19).

### Statistical analysis

Propensity score matching (PSM [1:2 nearest neighbor-matching with a caliper of 0.3]) method was performed to adjust for imbalances of patients’ characteristics between the two groups. Pearson chi-squared or Fisher’s exact tests were performed to compare categorical variables. Differences in continuous variables between groups were assessed with the Mann-Whitney U test. The OS, PFS and DoR were estimated Kaplan-Meier (log-rank test) method. All statistical analyses were performed with IBM SPSS 26.0 software (SPSS Inc., Chicago, IL) or the R language statistical software version 4.1.2. Two-tailed P values less than 0.05 (P < 0.05) were considered statistically significant.

## Results

### Patient and characteristics

After 1:2 PSM, 81 patients were enrolled (30 in the Anti-PD-1+CRT group and 51 in the CRT group), 9 (30.0%) patients are clinically assessed as inoperable and 21 (70.0%) patients or patients’ families are unwilling to operate in the Anti-PD-1+CRT group, 13 (25.5%) and 38 (74.5%) patients in the CRT group, respectively (**Supplementary Table 4**). The baseline characteristics and intervention factors were well-balanced (**Table 1**).

**Table 1:** Clinical characteristics of patients.

	Anti-PD-1+CRT (n = 30)	CRT (n = 51)	*P*-value
Male	20 (66.7)	40 (78.4)	0.243^a^
Age (range, years)	68 (47–74)	70 (50–75)	0.753^b^
<65	5 (16.7)	7 (13.7)	
≥65	25 (83.3)	44 (86.3)	
ECOG			0.654^a^
0	22 (73.3)	35 (68.6)	
1	8 (26.7)	16 (31.4)	
Smoking	5 (16.7)	13 (25.5)	0.356^a^
Chronic disease	7 (23.3)	10 (19.6)	0.691^a^
Histological grade			0.686^b^
Well differentiated	7 (23.3)	14 (27.5)	
Moderately differentiated	10 (33.3)	20 (39.2)	
Poorly differentiated	11 (36.7)	12 (23.5)	
Indeterminate	2 (6.7)	5 (9.8)	
Tumor location			0.505^b^
Cervical	1 (3.3)	1 (2.0)	
Upper thoracic	5 (16.7)	16 (31.4)	
Middle thoracic	16 (53.3)	21 (41.2)	
Lower thoracic	8 (26.7)	13 (25.5)	
AJCC ^8th^ stage			0.307^c^
II	6 (20.0)	14 (27.5)	
III	19 (63.3)	32 (62.7)	
IVa	5 (16.7)	5 (9.8)	
T stage			0.804^b^
T1	3 (10.0)	5 (9.8)	
T2	9 (30.0)	15 (29.4)	
T3	16 (53.3)	28 (54.9)	
T4	2 (6.7)	3 (5.9)	
N stage			0.738^b^
N0	3 (10.0)	7 (13.7)	
N1	10 (33.3)	18 (35.3)	
N2	13 (43.3)	23 (45.1)	
N3	4 (13.3)	3 (5.9)	
Chemotherapy regimen			0.26^a^
FP	18 (60.0)	24 (47.1)	
TP	12 (40.0)	27 (52.9)	
Chemotherapy cycle			
2 cycles	24 (80.0)	39 (76.5)	0.712^a^
3–4 cycles	6 (20.0)	12 (23.5)	
Radiation does (range, Gy)	50.4 (50.0–64.8)	50.4 (50.0–66.0)	0.239^c^
Anti-PD-1 antibody			
Tislelizumab	13 (43.3)		
Camrelizumab	10 (33.3)		
Pembrolizumab	7 (23.3)		

Unless otherwise indicated, all data are n (%); Anti-PD-1, anti-programmed cell death protein 1; CRT, chemoradiotherapy; ECOG, Eastern Cooperative Oncology Group; AJCC, American Joint Committee on Cancer; FP, fluoropyrimidine + platinum; TP, paclitaxol + platinum; Chronic disease: cardiovascular and pulmonary comorbidity. a: χ2 test; b: Fisher’s exact test; c: Mann-Whitney U test.

### Clinical follow-up

**Figure 2:**
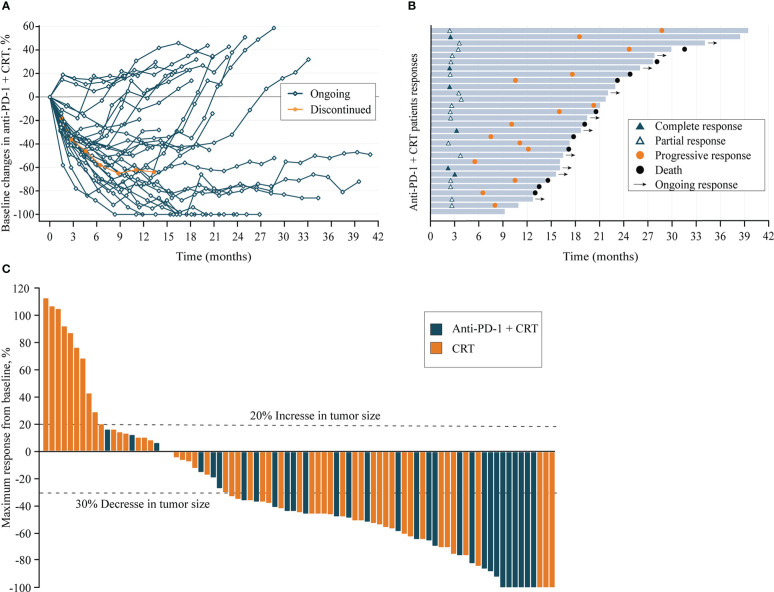
**(A)**, Longitudinal change in sum of longest target lesion diameters from baseline in Anti-PD-1+CRT group (n=30); **(B)**, Duration of exposure and response in Anti-PD-1+CRT group (n=30); **(C)**, Best change from baseline in sum of longest target lesion diameters per patient (n = 81). Anti-PD-1, anti-programmed cell death protein 1; CRT, chemoradiotherapy; Ongoing response: As of the last assessment (treatment ongoing), patient remains with partial response; Discontinued: Permanent cessation of treatment immunotherapy.

At the data cut-off date of 30 June 2022, the median duration of follow-up was 31.4 (95% CI, 22.2–40.6) months in all patients and 22.5 (95% CI,19.4–24.8) months in Anti-PD-1+CRT group patients, with 79 (97.5%) completed the follow-up assessment. All patients completed planned radiotherapy with a median dose of 50.4 Gy (range 50 to 66 Gy). Anti-PD-1+CRT group received at least 4 cycles of immunotherapy with full-dose intensity, the median of anti-PD-1 treatment was 6 cycles. During the study, 3 (10.0%) patients experienced interruptions in the Anti-PD-1+CRT group, including one patient in the permanently discontinued treatment due to severe immune-related myocarditis (**Figure 2A**). The most common reasons for discontinuation of treatment were disease progression (PD) or death, and severe irAEs in the Anti-PD-1+CRT group (**Figure 2B**).

### Antitumor activity

All patients had received at least one baseline radiographic assessment after radiotherapy (**Figure 2C**). The proportion of target lesion shrinkage in the Anti-PD-1+CRT group was significantly higher than CRT group (90.0% *vs.* 68.6%, P = 0.028). The data of the 81 response-evaluable patients are listed in **Table 2**. The CR and PR rate in Anti-PD-1+CRT and CRT groups was 20.0% *vs.* 5.9% and 60.0% *vs.* 51.0%, respectively, and the difference in efficacy distribution between the two groups was statistically significant (P = 0.027).

**Table 2:** Tumor response to treatment.

	Anti-PD-1+CRT (n = 30)	CRT (n = 51)	*P-*value
ORR^b^, n (%, 95% CI)	24 (80.0, 61.4–92.3)	29 (56.9, 42.2–70.7)	0.034
DCR^c^, n (%, 95% CI)	30 (100, 88.4–100)	42 (82.4, 69.1–91.6)	0.023
Best overall response, n (%)			0.027
CR	6 (20.0)	3 (5.9)	
PR	18 (60.0)	26 (51.0)	
SD	6 (20.0)	13 (25.5)	
PD		9 (17.6)	
mToR (range, months)	2.6 (2.2–3.9)	3.1 (2.1–4.5)	0.214
mDoR (range, months)	17.3 (4.7–30.7+)	11.1 (2.7–25.9)	0.022

Anti-PD-1, anti-programmed cell death protein 1; CRT, chemoradiotherapy; CI, confidence interval; CR, complete response; PR, partial response; SD, stable disease; PD, progressive disease; mToR, median time to response; mDoR, median duration of response; The + indicates that there was no progressive disease at last disease assessment. a: Confirmed by repeat radiographic assessment ≥ 4 weeks after first documentation of response; b: Complete response + partial response; c: Complete response + partial response + stable disease maintained for ≥ 2 months.

### Survival outcomes

**Figure 3:**
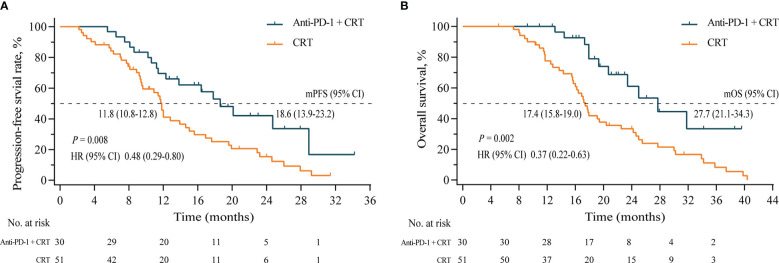
**(A)**, Progression-free survival of the Anti-PD-1+CRT group (n = 31) and the CRT group (n =51); **(B)**, Overall survival of two groups. Anti-PD-1, anti-programmed cell death protein 1; CRT, chemoradiotherapy; mPFS, median progression-free survival; mOS, median overall survival; HR, hazard ratio; CI, confidence intervals.

The median PFS of the Anti-PD-1+CRT group was 18.6 (95% CI, 13.9–23.2) months, nearly double that of the CRT group, which was 11.8 (95% CI, 10.8–12.8) months (HR= 0.48, P = 0.008) (**Figure 3A**). Likewise, the median OS of the Anti-PD-1+CRT group was significantly higher than that of the CRT group (27.7 [95% CI, 21.1–34.3] *vs.* 17.4 [95% CI, 15.8–19.0], HR 0.37 [95% CI, 0.22–0.63], P =0.002) months, with 1-year OS rate of 93.3% *vs.* 72.5%, respectively (P = 0.001) (**Figure 3B**).

The ORR and DCR of the Anti-PD-1+CRT group were higher than those of the CRT group, (80.0% *vs.* 56.9%, P = 0.034) and (100% *vs.* 82.4%, P = 0.023), respectively. In terms of response time, the median time to response and median duration of response (DoR) for the Anti-PD-1+CRT group was 2.6 (2.2–3.9) months and 17.3 (4.7–30.7+ [+, indicates that there was no progressive disease at last disease assessment]) months, respectively, whereas the median time to response and DoR for CRT group was 3.1 (2.1–4.5) months and 11.1 (2.7–25.9) months, respectively (**Table 2**).

### Treatment toxicity

Both treatment schedules were generally well tolerated. The incidence of all grades of AEs in the Anti-PD-1+CRT group and CRT group were 93.3% (28 of 30) and 92.2% (47 of 51) (P = 0.845), and the incidence of ≥ grades 3 was 50.0% (15 of 30) and 33.3% (17 of 51) (P = 0.138). The most common AEs ≥ grade 3 of the two regimens are hematological toxicity (lymphocytopenia, leukopenia, and neutropenia), and hematological toxicity of grade 3 or above occurred in 40.0% (12 of 30) of Anti-PD-1+CRT group and 27.5% (14 of 51) of CRT group (P = 0.243). Moreover, the occurrence of lymphocytopenia toxicities was greater in Anti-PD-1+CRT than CRT (60.0% *vs.* 23.5%, P = 0.01). In terms of non-hematological AEs (fatigue, rash, nausea/vomiting, diarrhea, hypoalbuminemia, abnormal liver function, peripheral neuropathy, esophageal perforation, pneumonia), and the incidence of pneumonia (33.3% *vs.* 13.7%, P = 0.036) in Anti-PD-1+CRT group was higher than that in CRT group. 43.3% of patients (13 of 30) experienced irAEs in the Anti-PD-1+CRT group, including one patient who permanently discontinue treatment for grade 3 auto-immune myocarditis and died of a severe lung infection 4 months later. For delayed toxic reactions ≥ 6 months, the incidence of abnormal renal function in the Anti-PD-1 + CRT group and the CRT group was 6.7% and 2.0%, respectively (P= 0.552). In the Anti-PD-1+CRT group, one patient with chronic obstructive pulmonary disease (COPD) developed grade 2 pneumonia 7 months after receiving anti-PD-1. Anti-PD-1 therapy was restarted again when the irAEs of the rest patients were improved by corticosteroids to grade 1 or lower, and no patients had recurrence of grade ≥ 2 irAEs (**Table 3**).

**Table 3:** Incidence of Treatment-related toxicities.

	Any Grade^a^		*P-*value	≥ Grade 3		*P-*value
	Anti-PD-1 +CRT (n = 30)	CRT (n = 51)		Anti-PD-1 +CRT (n = 30)	CRT (n = 51)	
Treatment-related AEs^b^	28 (93.3)	47 (92.2)	0.845	15(50.0)	17 (33.3)	0.138
Hematological toxicity	24 (80.0)	39 (76.5)	0.712	12 (40.0)	14 (27.5)	0.243
Lymphocytopenia	18 (60.0)	12 (23.5)	0.01	8 (26.7)	6 (11.8)	0.087
Leukopenia	13 (43.3)	15 (29.4)	0.203	5 (16.7)	7 (13.7)	0.753
Neutropenia	11 (36.7)	17 (33.3)	0.761	4 (13.3)	9 (17.6)	0.758
Anemia	8 (26.7)	11 (21.6)	0.601			
Thrombocytopenia	7 (23.3)	8 (15.7)	0.392	3 (10.0)	3 (5.9)	0.665
Fatigue	15 (50.0)	23 (45.1)	0.669	4 (13.3)	4 (7.8)	0.46
Rash	8 (26.7)	10 (19.6)	0.461			
Nausea/vomiting	6 (20.0)	8 (15.7)	0.62		2 (3.9)	0.528
Diarrhea	4 (13.3)	5 (9.8)	0.72	1 (3.3)		0.892
Hypoalbuminemia	6 (20.0)	10 (19.6)	0.996	2 (6.7)	3 (5.9)	0.887
Abnormal liver function	5 (16.7)	7 (13.7)	0.753	2 (6.7)	2 (3.9)	0.624
Peripheral neuropathy	4 (13.3)	6 (11.8)	0.836			
Esophageal perforation	1 (3.3)	3 (5.9)	0.609			
Pneumonia	10 (33.3)	7 (13.7)	0.036			
Immune-related AEs^c^	13 (43.3)					
Reactive capillary endothelial proliferation	5 (16.7)					
Enteritis	4 (13.3)					
Myocarditis	3 (10.0)			1 (3.3)		
Hypothyroidism	3 (10.0)					
Hyperthyroidism	1 (3.3)					
Late toxicity (≥6 months)						
Abnormal renal function	2 (6.7)	1 (2.0)	0.552			
Pneumonitis^d^	1 (3.3)					

Unless otherwise indicated, all data are n (%); AEs, adverse events; Anti-PD-1, anti-programmed cell death protein 1; CRT, chemoradiotherapy. a: AEs were classified according to NCI common terminology for adverse events version 5.0; b: Treatment-related AEs occurring in 5% or more of patients in either group are listed; c: Immune–related AEs occurring in one or more of patients in Anti-PD-1+CRT group are listed; d: Pneumonitis was observed as fibrosis or consolidation occurring in the previous radiation therapy field.

## Discussion

In this real-world study, compared with 11.8 months in the CRT group, the median PFS in the Anti-PD-1+CRT group was prolonged to 18.6 months (HR = 0.48, P = 0.008), and also translated to OS benefits (median 27.7 *vs.* 17.4 months, HR 0.37, P = 0.002). In terms of ORR, the Anti-PD-1+CRT group was significantly better than the CRT group (80.0% *vs.* 56.9%, P = 0.034). A higher target lesion shrinkage rate (90.0%) was observed in the Anti-PD-1+CRT group, 6 (20.0%) patients were evaluated as CR, while only 3 (5.9%) patients were in the CRT group. Compared with the CRT group, although the Anti-PD-1+CRT group had no significant difference in response time (2.6 *vs.* 3.1 months), the DoR of the Anti-PD-1+CRT group was longer (median 17.3 vs. 11.1 months, P = 0.022). These results show that, compared with chemoradiotherapy alone, Anti-PD-1 plus chemoradiotherapy has the potential for sustained remission and disease control in patients with locally advanced ESCC. Since this study aimed at using PD-1 inhibitors concurrently with concurrent radiotherapy, clinicians were initially worried that side effects would aggravate. Our results showed that the incidence of AEs was not statistically significant between the two groups (93.3% *vs.* 92.2%, P = 0.845), and no additional toxic effects occurred compared with previous studies (20, 21).

In this study, Ren’s standard was used in combination with RECIST1.1 (or irRECEST) criteria to evaluate the efficacy, because of the limitations of the application of RECIST1.1 (or irRECEST) criteria in such a hollow organ as the esophagus. In Ren’s study, CT combined with esophagography was used to measure the thickness of esophageal wall and the short diameter of lymph nodes to evaluate the efficacy (18). Some previous studies have found that tumor immunotherapy may potentially delay benefit, and the irRECEST standard also recommends that new tumor lesions that have not been evaluated as disease progression after immunotherapy should be re-evaluated after 8 weeks of immunotherapy to rule out the possibility of false progression (17, 22). In this study, we did not observe patients with delayed remission or false progression, which may be related to enhanced local control by radiotherapy.

Currently, adding anti-PD-1 to CRT is not the standard treatment for locally advanced esophageal squamous cancer (ESCC). KEYNOTE-590 and CHECKMATE-648 phase III trials demonstrated good survival benefits of anti-PD-1 in combination with chemotherapy for the first-line treatment of advanced unresectable/metastatic ESCC. Recently, PD-1 inhibitors Camrelizumab, Sintilimab, Toripalimab, and Tislelizumab combined with chemotherapy have successively shown survival benefits and manageable toxicity in the first-line treatment of patients with advanced unresectable/metastatic ESCC in China (23–26). These drugs are more cost-effective due to China’s health insurance policy. In patients with locally advanced esophageal cancer, despite aggressive concurrent chemoradiotherapy regimens, survival was suboptimal, with the majority of patients experiencing progression. In the real world, many patients with locally advanced ESCC are treated with immunotherapy combined with concurrent chemoradiotherapy after clinicians have obtained informed consent from them and their families, in the hope of improving their survival. This study analyzed real data from three clinical centers, and the results showed that the OS and PFS in the CRT group were similar to those in previous large sample studies (27–29). Phase III studies KEYNOTE-975, KUNLUN, and RATIONALE 311 are also underway for the use of chemoradiotherapy in combination with immunotherapy in locally advanced unresectable ESCC on the International Clinical Trials Registry Platform (ICTRP) (30–32).

The real-world study design has inherent limitations on the data available for analysis. Firstly, the expression of PD-L1, microsatellite instability-high or defective mismatch repair (MSI-H/dMMR) in patients has not been tested. Secondly, the heterogeneity of chemoradiotherapy regimens in this study, including chemotherapy regimens and different doses of radiotherapy. Thirdly, the overall sample size is small, so subgroup analysis is not allowed.

In conclusion, anti-PD-1 plus chemoradiotherapy has shown encouraging antitumor activity and tolerable safety in real-world circumstances. These results represent an important step forward in offering a viable treatment option for patients with locally advanced ESCC.

## Data availability statement

The datasets presented in this study can be found in online repositories. The names of the repository/repositories and accession number(s) can be found in the article/**Supplementary Material**.

## Ethics statement

The studies involving human participants were reviewed and approved by the Ethics Committee of Affiliated Hospital of Xuzhou Medical University. The patients/participants provided their written informed consent to participate in this study.

## Author contributions

JM, NY, and JL all have substantial contributions to the collection and analysis of data. ZQ and YY conceived and designed the study. The rest of the authors have given substantial contributions to the work by providing editing and writing assistance. All authors vouch for the respective data and analysis, approved the final version and agreed to submit the manuscript.
