# Supplementary material for: Efficacy and safety of anti-PD-1 antibody plus chemoradiotherapy in locally advanced esophageal squamous cancer
Source: Front Oncol. 2023 Feb 9;13:1005856. doi: 10.3389/fonc.2023.1005856 (PMC9947779; doi:10.3389/fonc.2023.1005856)
Supplement: Supplementary file 1 [file Table_1.docx]

**Supplementary**

**Table 1** Efficacy evaluation criteria of treatment in this study

|  | | CR | PR | | | | SD | PD^*^ |
| --- | --- | --- | --- | --- | --- | --- | --- | --- |
|  |  |  | i | ii | iii | iv |  |  |
| Esophageal lesion | Evaluation of barium meal examination | The tumor disappears completely, the edges of the esophagus are smooth, the barium passes smoothly, but the wall may be slightly straightened, the lumen is not narrowed or slightly narrowed (the ratio of the normal part of the upper esophagus to the narrowed part of the lesion is ≥3:2), if the mucosal image is shown, the mucosa is basically normal again or thickened. | The tumor disappears completely, the edges of the esophagus are smooth, the barium passes smoothly, but the wall may be slightly straightened, the lumen is not narrowed or slightly narrowed (the ratio of the normal part of the upper esophagus to the narrowed part of the lesion is ≥3:2), if the mucosal image is shown, the mucosa is basically normal again or thickened. | The tumor disappears completely, the edges of the esophagus are smooth, the barium passes smoothly, but the wall may be slightly straightened, the lumen is not narrowed or slightly narrowed (the ratio of the normal part of the upper esophagus to the narrowed part of the lesion is ≥3:2), if the mucosal image is shown, the mucosa is basically normal again or thickened. | Most of the lesion has disappeared, there is no obvious distortion or angulation, there is no extra-luminal ulceration, the barium passage is still smooth, but the margins are not smooth, there are small filling defects and/or niche shadows, or the margins are smooth but there is significant narrowing of the lumen (the ratio of the normal lumen at the upper end of the lesion compared to the narrowing of the lesion is less than 3:2). | Most of the lesion has disappeared, there is no obvious distortion or angulation, there is no extra-luminal ulceration, the barium passage is still smooth, but the margins are not smooth, there are small filling defects and/or niche shadows, or the margins are smooth but there is significant narrowing of the lumen (the ratio of the normal lumen at the upper end of the lesion compared to the narrowing of the lesion is less than 3:2). | Neither sufficient decrease in the sum of diameters to qualify for PR nor sufficient increase to qualify for PD. | Significant worsening of esophageal strictures, with still significant filling defects and/or niche shadows  (Judged by the researcher). |
|  | CT evaluation of maximum wall thickness | ≤12mm | ≤12mm | >12mm | ≤12mm | >12mm |  | >12mm (Esophageal lesion wall thickness increased by at least 20%, using the previous minimum wall thickness as reference.) |
| Regional lymph nodes |  | None/Short diameter of residual lymph nodes ≤ 10mm | Short diameter of target lymph nodes > 10mm (At least a 30% decrease in the sum of the diameters of target lymph nodes lesions, taking as reference the baseline sum of diameters). Non-target lymph nodes lesions didn't progress. | None/Short diameter of residual lymph nodes ≤ 10mm | None/Short diameter of residual lymph nodes ≤ 10mm | None/Short diameter of residual lymph nodes ≤ 10mm |  | At least a 20% increase in the sum of diameters of target lymph node lesions, taking as reference the smallest previous sum of diameters. In addition to the relative increase of 20%, the sum must demonstrate an absolute increase of at least 5 mm from nadir (or appearance of new enlarged pathological regional lymph nodes) |
| Distant metastasis |  | None | None | None | None | None | None | One or more |

CR: complete response; PR: partial response; SD: stable disease; PD: progression of disease. *: Only one of these needs to be met to be assessed as PD.

**Table 2** RECIST 1.1^*^ criteria concise version

| Evaluation of target lesions | | | |
| --- | --- | --- | --- |
| Complete response (CR) | | Disappearance of all target lesions. Any pathological lymph nodes (whether target or non-target) must have reduction in short axis to <10 mm. | |
| Partial response (PR) | | At least a 30% decrease in the sum of diameters of target lesions, taking as reference the baseline sum diameters. | |
| Stable disease (SD) | | Neither sufficient decrease in the sum of diameters to qualify for PR nor sufficient increase to qualify for progression of disease, taking as reference the smallest sum diameters while on study. | |
| Progression of disease (PD) | | At least a 20% increase in the sum of diameters of target lesions, taking as reference the smallest sum on study (this includes the baseline sum if that is the smallest on study). In addition to the relative increase of 20%, the sum must also demonstrate an absolute increase of at least 5 mm. (Note: the appearance of one or more new lesions is also considered progression). | |
| Evaluation of non-target lesions | | | |
| CR | | Disappearance of all non-target lesions and normalization of tumor marker level. All lymph nodes must be non-pathological in size (<10 mm short axis). | |
| Non-CR/Non-PD | | Persistence of one or more non-target lesion(s) and/or maintenance of tumor marker level above the normal limits. | |
| PD | | Unequivocal progression of existing non-target lesions. (Note: the appearance of one or more new lesions is also considered progression). | |
| Overall visit response | | | |
| Target lesions | Non-target lesions | New lesions | Overall response |
| CR | CR | No | CR |
| CR | Non-CR/non-PD | No | PR |
| PR | Non-PD | No | PR |
| SD | Non-PD | No | SD |
| PD | Any | Yes or No | PD |
| Any | PD | Yes or No | PD |
| Any | Any | Yes | PD |

CR: complete response; PD: progression of disease; PR: partial response; SD: stable disease. *: This table is from Response Evaluation Criteria in Solid Tumors, Version 1.1 (RECIST 1.1) guidelines (Eisenhauer et al 2009).

**Table 3** Criteria for evaluating the efficacy of treatment for esophageal cancer (Ren's study)

| Evaluation indicators | CR | PR | | | | NR/PD |
| --- | --- | --- | --- | --- | --- | --- |
|  |  | i | ii | iii | iv |  |
| Primary lesion |  |  |  |  |  |  |
| Evaluation of barium meal examination after treatment | The tumor disappears completely, the edges of the esophagus are smooth, the barium passes smoothly, but the wall may be slightly straightened, the lumen is not narrowed or slightly narrowed (the ratio of the normal part of the upper esophagus to the narrowed part of the lesion is ≥3:2), if the mucosal image is shown, the mucosa is basically normal again or thickened. | The tumor disappears completely, the edges of the esophagus are smooth, the barium passes smoothly, but the wall may be slightly straightened, the lumen is not narrowed or slightly narrowed (the ratio of the normal part of the upper esophagus to the narrowed part of the lesion is ≥3:2), if the mucosal image is shown, the mucosa is basically normal again or thickened. | The tumor disappears completely, the edges of the esophagus are smooth, the barium passes smoothly, but the wall may be slightly straightened, the lumen is not narrowed or slightly narrowed (the ratio of the normal part of the upper esophagus to the narrowed part of the lesion is ≥3:2), if the mucosal image is shown, the mucosa is basically normal again or thickened. | Most of the lesion has disappeared, there is no obvious distortion or angulation, there is no extra-luminal ulceration, the barium passage is still smooth, but the margins are not smooth, there are small filling defects and/or niche shadows, or the margins are smooth but there is significant narrowing of the lumen (the ratio of the normal lumen at the upper end of the lesion compared to the narrowing of the lesion is less than 3:2). | Most of the lesion has disappeared, there is no obvious distortion or angulation, there is no extra-luminal ulceration, the barium passage is still smooth, but the margins are not smooth, there are small filling defects and/or niche shadows, or the margins are smooth but there is significant narrowing of the lumen (the ratio of the normal lumen at the upper end of the lesion compared to the narrowing of the lesion is less than 3:2). | There is residual lesion or no visible improvement in the lesion, and there are significant filling defects and/or niche shadows. |
| CT evaluation of maximum wall thickness | ≤1.2cm | ≤1.2cm | >1.2cm | ≤1.2cm | >1.2cm | Any |
| Regional lymph nodes | None/Short diameter of residual lymph nodes ≤ 1.0cm | Short diameter of residual lymph nodes > 1.0cm | None/ No enlargement of lymph nodes after treatment | None/ No enlargement of lymph nodes after treatment | None/ No enlargement of lymph nodes after treatment | Any |
| Distant metastasis | None | None | None | None | None | One or more |

CR: complete response; PR: partial response; NR: no response; PD: progression of disease.

**Table 4:** Clinical features of patients before PSM

|  | Anti-PD-1+CRT  (n = 38) | CRT  (n = 85) | *P*-value |
| --- | --- | --- | --- |
| Male | 24 (63.2) | 67 (78.8) | 0.067 |
| Age (range, years) | 68 (47–75) | 71 (50–75) | 0.01 |
| <65 | 8 (21.1) | 12 (14.1) |  |
| ≥65 | 30 (78.9) | 73 (85.9) |  |
| ECOG |  |  | 0.258 |
| 0 | 26 (68.4) | 36 (42.4) |  |
| 1 | 12 (31.6) | 49 (57.6) |  |
| Smoking | 6 (15.8) | 23 (27.1) | 0.174 |
| Chronic disease | 11 (28.9) | 18 (21.2) | 0.348 |
| Histological grade |  |  | 0.637 |
| Well differentiated | 9 (23.7) | 20 (23.5) |  |
| Moderately differentiated | 14 (36.8) | 27 (31.8) |  |
| Poorly differentiated | 12 (31.6) | 24 (28.2) |  |
| Indeterminate | 3 (7.9) | 14 (16.5) |  |
| Tumor location |  |  | 0.434 |
| Cervical | 1 (2.6) | 2 (2.4) |  |
| Upper thoracic | 6 (15.8) | 25 (29.4) |  |
| Middle thoracic | 21 (55.3) | 37 (43.5) |  |
| Lower thoracic | 10 (26.3) | 21 (24.7) |  |
| AJCC ^8th^ stage |  |  | 0.145 |
| II | 7 (18.4) | 26 (30.6) |  |
| III | 24 (63.2) | 52 (61.2) |  |
| IVa | 7 (18.4) | 7 (8.2) |  |
| Chemotherapy regimen |  |  | 0.081 |
| FP | 23 (60.5) | 37 (43.5) |  |
| TP | 15 (39.5) | 48 (56.5) |  |
| Radiation does (range, Gy) | 50.4 (50.0–64.8) | 50.4 (50.0–66.0) | 0.153 |

Unless otherwise indicated, all data are n (%); Anti-PD-1: anti-programmed cell death protein 1; CRT: chemoradiotherapy; ECOG: Eastern Cooperative Oncology Group; AJCC: American Joint Committee on Cancer; FP: fluoropyrimidine + platinum; TP: paclitaxol + platinum; Chronic disease: cardiovascular and pulmonary comorbidity.

# References

1. Eisenhauer EA, Therasse P, Bogaerts J, Schwartz LH, Sargent D, Ford R, et al. New response evaluation criteria in solid tumors: revised RECIST guideline (version 1.1). Eur J Cancer 2009;45(2):228-47.

2. Ren XJ, Xu lA, Wang l, Chen ll, Han C. Clinical application of barium radiography and computed tomography-based short-term outcome evaluation criteria for esophageal cancer. Chinese Journal of Radiation Oncology. 2018;27(5):6. doi:10.3760/cma.j.issn.1004-4221.2018.05.004.
